# Insights on biology student motivations and challenges when reading and analyzing primary literature

**DOI:** 10.1371/journal.pone.0251275

**Published:** 2021-05-10

**Authors:** Kristen N. Howard, Emma K. Stapleton, April A. Nelms, Kelsee C. Ryan, Miriam Segura-Totten

**Affiliations:** 1 Department of Water Resources, City of Gainesville, Gainesville, Georgia, United States of America; 2 Johnson High School, Gainesville, Georgia, United States of America; 3 College of Education, University of North Georgia, Dahlonega, Georgia, United States of America; 4 Department of Biological Sciences, Clemson University, Clemson, South Carolina, United States of America; 5 Department of Biology, University of North Georgia, Dahlonega, Georgia, United States of America; University of Sydney, AUSTRALIA

## Abstract

Reading primary literature is a popular classroom practice that exposes students to the process of science. However, the analysis of primary literature can be taxing and time-consuming for students. For this reason, it is important to determine the source of student challenges and what motivates them to read primary literature. To better understand students’ challenges, preferences, and motivations towards analyzing primary literature, we held focus groups with biology undergraduates where we asked them about their thoughts and perceptions on this practice. Students felt they struggle with understanding the big picture of an article, certain aspects of scientific literacy like data interpretation and experimental setup, and lack of knowledge of terms and techniques. Further analysis of the data using the achievement goal and expectancy-value theories of motivation revealed that students: 1) demonstrate mastery and performance approach goal orientations, which are typically associated with positive learning outcomes, 2) value the usefulness of reading primary literature, and 3) feel most engaged in the process of reading an article when the topic interests them. We provide pedagogical recommendations based on our findings.

## Introduction

Scientific reasoning and critical thinking skills are nationally recognized as necessary for undergraduates’ college and career readiness [1, 2]. Reading and analyzing primary literature boosts these two skills and the understanding of the process of science [3–7]. Many approaches for analyzing research papers in the sciences have been developed, tested, and published (e.g., [8–18]). However, reading primary literature is a time-consuming and complex task that demands focus and can cause students frustration. Thus, it remains a challenge to engage students with the primary literature in a productive way which does not heighten frustration and anxiety.

Students devote more time to a learning task, and learning occurs more effectively when they are motivated to engage with the learning process [19–21]. Thus, motivation can act as an intrinsic driver for students to connect with classroom material [19, 22]. There are also extrinsic factors that can motivate students, such as graded assignments. A meta-analysis of studies that looked at the effects of performance-contingent rewards (i.e., rewards based on how well the participant does on a task, such as graded work) suggests that these extrinsic ways of motivating students can lead to decreased intrinsic motivation [23]. However, we have found that giving students reading assignments prior to discussing a research article is important for them to engage deeply with the text [5]. These findings highlight the need for instructors to strike a balance between fostering intrinsic motivation and extrinsically motivating students to engage productively with a complex task like reading primary literature.

**Fig 1:**
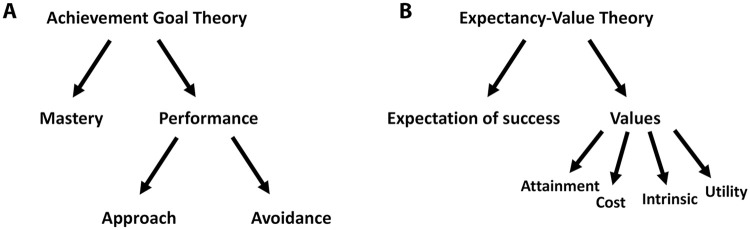
Schematic of the achievement goal and expectancy-value theories of motivation. A. According to the achievement goal theory, students can be motivated by becoming proficient in certain academic tasks (Mastery), or by how they perform relative to their peers (Performance). Students can be motivated either by performing better than their peers (Approach) or by fear of failure (Avoidance). B. The expectancy-value theory states that expectancy (Expectation of success) and certain characteristics related to the task (Values; Attainment, Cost, Intrinsic, and Utility) drive students to engage in a specific task.

Several theoretical frameworks help to explain the factors that influence student motivation [24–33]. Different frameworks for student motivation are not mutually exclusive and can be used together to describe student behavior in the classroom [34]. Two widely recognized frameworks are the achievement goal and expectancy-value theories. Achievement goal theory states that students can be motivated by either becoming competent in certain academic tasks (i.e., mastery goals) or by measuring their performance relative to their peers (i.e., performance goals [25, 27]). Students can adopt a performance approach goal, where they strive to do better than their peers, or a performance avoidance goal, where they are motivated by fear of failure [35]. While mastery and performance approach goals are generally associated with positive outcomes, such as feeling successful and increased persistence regardless of perceived intellectual ability, performance avoidance goals can be associated with negative outcomes [34, 36], reviewed in [37]. The expectancy-value theory of motivation focuses on the values that drive students to pursue a specific classroom task and the role that the expectation of future success plays in student motivation [24]. The four task values described by the theoretical framework are: 1) attainment, or the importance the individual assigns to doing well in a task because of how the task relates to their identity; 2) cost, which encompasses the perceived negative effects associated with the task; 3) intrinsic, which describes the individual’s innate interest in the task; and 4) utility, or how the task contributes to the individual’s future goals. We summarize the main components of these frameworks in Fig 1.

Scientific primary literature has a high intrinsic cognitive load; that is, articles contain highly detailed information, such as experimental methods and technical vocabulary. This can easily overload the short-term memory of undergraduates who do not know these terms and techniques since an individual’s short-term memory can only hold a limited number of facts [38, 39]. This might in turn lead students to lose interest in reading a research article. As individuals progress in their studies, the new information they come across is eventually organized into informational schemas and stored in long-term memory [38, 40]. This partly explains why PhD-level scientists can better grapple with the information in primary literature compared to undergraduates [41–43].

Students’ motivation plays a role in how engaged they are with a learning task. Moreover, positive aspects of motivation can enhance persistence in science [44]. This might be because students who are motivated exhibit constructive behaviors that lead to positive outcomes in science courses [22]. For this reason, we wanted to understand what motivates students to read the primary literature. Additionally, we were interested in: 1) determining the challenges students face when analyzing primary literature, and 2) investigating students’ opinions on how instructors can promote productive learning during the reading of primary literature. Specifically, our research questions were:

RQ_1_: What motivates biology students to read primary literature?
RQ_2_: What challenges do biology students face when they read primary literature?
RQ_3_: What are biology students’ perceptions of strategies instructors can use to facilitate reading primary literature?

To investigate our research questions, we held focus groups to obtain students’ perceptions about reading and analyzing primary literature. We found that students in our study feel they struggle with understanding the big picture of an article, certain aspects of primary literacy like data interpretation and experimental setup, and lack of knowledge of terms and techniques. Our data also reveal that students realize the value of reading primary literature, even if they find this practice arduous. In addition, students in our study demonstrated positive goal orientations toward the practice of literature analysis. They were also more likely to be deeply engaged with a research article on a topic of their interest. Our results suggest ways in which instructors can structure the classroom to increase student engagement and enjoyment of reading primary literature.

## Methods

### Context

The study was conducted at a four-year, master’s degree granting regional university in the southeastern United States. The student population is approximately 20,000 across five campuses. The focus groups were conducted in the College of Science and Mathematics on the university’s only residential undergraduate campus. Approval for this study (expedited status, application 2014116) was granted by the Institutional Research Board at the University of North Georgia.

### Participants

To identify student participants, MS-T emailed students enrolled in coursework in the biology department to provide them with the opportunity to participate. Participation in the study was completely voluntary. MS-T provided students wishing to volunteer with additional study information and the informed consent form. Of the 11 students who chose to participate in the focus groups, 9 were biology majors, and two were identified as business majors. One of these two individuals was pursuing a biology minor, and the other had taken the introductory biology course sequence. Our study population was representative of the university biology student population in terms of ethnicity, gender and GPA, except for the underrepresentation of African American students (Table 1). While Asian, Native Hawaiian, and Pacific Islander students appear over-represented in our sample (Table 1), the percentage indicated corresponds to one student out of the total number of participants. Thus, we have captured this student population as accurately as possible given the small sample size of the study. The unpaired t-test comparing GPA scores was performed using the free Graph Pad platform (www.graphpad.com). Study participants had varied levels of experience reading primary literature, with freshmen and sophomores having read 1–5 articles and juniors and seniors having read 12–50 articles (Table 1). While we do not have comparable data on articles read for the rest of the biology major population, the data from the study population align with our anecdotal observations that, as students progress through the biology program, they read more research articles in courses and as part of their involvement in research. As an additional note about the study participants, nine of the students simultaneously volunteered to participate in a separate but related study [45].

**Table 1:** Comparison of demographic characteristics of the study population and all biology undergraduates.

Population	GPA	Articles read	Ethnicity	Gender
**Study**	3.22 ± 0.17*	Freshmen and sophomores: 1–4	91% White	56% Female
			9% Latinx	44% Male
			9% Asian, Native Hawaiian, and Pacific Islander	
		Juniors and seniors: 12–50		
**All biology undergraduates**	3.08 ± 0.02*	N/A	84.4% White	56% Female
			11% Latinx	44% Male
			2.8% Asian, Native Hawaiian, and Pacific Islander	
			4.6% African	
			American	

GPA scores are shown with standard error of the mean (SEM).

*p = 0.4010.

### Data collection

MS-T, KNH, KCR, and EKS led 11 participants through two small-group facilitated discussions where students answered the questions shown in Table 2. The questions prompted students to discuss their general feelings, identify their positive and negative thoughts, identify the educational benefits, and express their frustrations regarding reading primary literature. In addition, we designed questions to determine how professors could help students comprehend the content or better enjoy the reading experience. One focus group included seven junior and senior biology majors; this group yielded 46 minutes of discussion and 20 pages of transcript material. The other focus group consisted of four first- and second-year students; their discussion lasted about 15 minutes and resulted in 6 pages of transcript material.

**Table 2:** Focus group discussion questions.

Questions
How do you feel about reading scientific articles and why do you feel this way? What do you enjoy and what do you dislike about reading scientific articles?
When reading scientific articles, if you find yourself confused or lost, why do you feel this way?
What is the greatest difficulty or frustration, if any, that you experience when reading a scientific article?
What can your professor do to facilitate your understanding or enjoyment of a scientific article?
What do you think is the most beneficial aspect of reading scientific articles?

### Data analysis

Initially, MS-T, KNH, and EKS analyzed the focus group transcripts using the constant comparative method [46] and an inductive coding process [47]. We first read the transcripts individually to categorize the text. After forming the initial list of codes, we met to discuss our work and what we understood each code to mean. Using the collaboratively developed code list, we analyzed the transcripts individually through focused coding until we were unable to produce new codes. We met as a group to discuss our coding and resolve inconsistencies until there was complete agreement between all coders, and we established consensus validation by avoiding double coding instances. While simultaneously coding the focus group data, MS-T coded data from think-aloud interviews of students and faculty reading through a research article [45]. We discuss this to bring transparency to our work because our analysis of the think-aloud data informed the coding of the focus group data. We realized we were finding common codes and decided to use the same code names across studies for clarity and consistency. This led to a final review of the data through thematic coding by MS-T, which resulted in our final list of 16 codes presented in Table 3. The code names that were revised to align with the think-aloud interviews were: cognitive load, jargon, lack of knowledge, scientific literacy, and wording/sentence structure. The codes were not defined differently; instead, the nomenclature was clarified when the same occurrences were observed in the data.

**Table 3:** Codebook developed from the analysis of focus group discussions.

*Code*	*Definition*	*Sample quote*
Big picture	Mentioned the overall idea of the article or of a section of the article.	The teacher summarizes it in a couple sentences before you read it like, this is about bioluminescence and this is a couple of things related then you’re not as completely clueless going into it. That’s what Dr. [name redacted] did for us and it helped me.
Cognitive load	Commented on foreign concepts within articles that make comprehension more difficult.	I think it’s uh, it’s uh I don’t know how to say this, like a burden or a load. So one ambiguous term is digestible, you can look it up. Two, you can manage it, but if you have nineteen ambiguous terms and then you’ve got nine graphs that still have five mentions of multivariable data. As opposed to one graph that’s silly, but at least you can battle that one silly graph.
Dislike certain assessments	Noted the dislike for some course assessments associated with reading scientific articles.	I don’t like answering a bunch of questions about [the article] ‘cause … it becomes less about like, trying to understand the article by looking [at] what the question’s [asking].
Group discussions	Mentioned in-class discussion of scientific articles.	Yeah, I do enjoy discussing the questions with the class. I feel like when you read it you don’t have the same viewpoint as other people.
Hold students accountable so they read article	Ways in which professors ensured students read the article prior to class discussion.	But Dr. [name redacted] actually made us go over everything to the class, with the graphs and stuff. I feel like that really helped, cause then you actually did have to know what the graphs meant. So yeah, I thought that was really cool.
Jargon	Mention of technical language that is unknown to participants.	I think initially, when I first started reading papers here, I just hated it because it was just, like, really, like super hard words.
Increases knowledge of topic	Mentioned that reading scientific articles increases knowledge of the topic.	It increases your knowledge…
Lack of confidence	Demonstrated a lack of confidence in reading and analyzing articles.	I like to, I like to read the graphs. I mean, just like, try to analyze them a little bit, but I’m just really not good at it, but I just like to do it.
Lack of knowledge	Noted lack of knowledge necessary to comprehend the article.	And so, it might be your genus and species of mice or it might be the technique or it might be the stats that they use. So if I look for those first and if something is really wacky and makes no sense I have to go and look it up first, I’ll do that.
Outside resources to aid understanding	Mentioned resources external to the article that help understanding.	Or like, with bioluminescence we had … a video I think that she … added on that would help you like, understand what was going on because it’s … one thing to read about it, but it’s another thing to actually see it to understand it.
Reading more articles is beneficial	Indicated that having more practice reading scientific articles is helpful.	I’d also say, kinda like, like I know it would be probably more annoying for students, but like, I’ve only ever had one teacher that’s ever had us do anything with a scientific article, so that if you only have one then it’s just kind of like, ‘oh my gosh, what do I do?’ But if you had more than, I mean, it would be like, a pain, but you’d get used to it and you’d be better with them.
Real life application	Commented on how reading articles highlights the real-life applications of science.	A lot of, a lot of my classmates and peers have a certain attitude, especially in physics. People bang their head on the wall and say organic, I’m never going to use this, I’m never going to use physics, I don’t care about genetics. You know, then you read these papers and then well, if you don’t have an understanding of physics and organic chemistry, you can’t set up any protocol, you can’t do anything. And that shows that all the stuff you have to tertiarily understand is vitally crucial, and it’s not just they’re forcing you to get more credit hours.
Scientific literacy	Mentioned an aspect of scientific literacy. We define scientific literacy as the skills related to 1) recognition and analysis of methods that lead to scientific knowledge, and 2) the organization, interpretation and analysis of scientific information [48].	Graphs are sometimes great if you understand them. But some of them, like that one I came to you with, um, from Dr. [name redacted]. I was like, I have no idea what this is trying to tell me, but all it was showing was variance.
Time investment	Commented on the amount of time necessary to read a research article.	The amount of time I have to put into [reading an article]. Like these are like five pages that should take me twenty minutes, thirty minutes but it’s taking me two hours when you have to find everything, highlight everything, and like figure it out.
Topic of article matters	Note getting more out of reading an article on a topic of interest.	Well, if I’m not, like, interested in the topic then I usually don’t like it ‘cause, there’s … a bunch of facts and facts …that … you don’t really care about or know about it’s just like, what’s the point of this? But when you’re interested in it, it’s nice to know what you’re reading about and I want to know what that means and like, you’re kind of into it and like, figuring it out so it’s kind of fun.
Wording/sentence structure	The writing style of the article prevented comprehension.	Sometimes it like gets too jumbled where it puts parenthesis trying to explain it, but then it’s got like, so much parenthesis running around I get confused.

The codes resulting from the analysis are shown, along with the definition and a sample quote. Codes are ordered alphabetically.

MS-T conducted further analysis of the focus group transcripts based on the achievement goal and expectancy-value theoretical frameworks. First, student comments were classified into mastery or performance orientations following the definitions of these dimensions of achievement goal theory [27]. Then, student comments were classified into one of the four task values within the expectancy-value theoretical framework: attainment, cost, intrinsic, and utility, following their established definitions [49]. We did not find student comments that were clearly related to the expectancy construct of the framework. We then quantified the total number of instances and the percentage of the total for each dimension.

## Results

In this study, we conducted focus groups to determine student motivations and challenges associated with reading primary literature. We first describe students’ perceptions about what they find challenging when reading primary literature and how they think instructors can help them succeed at this task. Then, we report our findings in light of the achievement goal and expectancy-value theories of motivation.

### Student perceptions of their experience reading primary literature

Students reported having issues establishing the overall idea or “big picture” of an article. For example, when asked about their likes and dislikes when reading the literature and why they feel confused or frustrated when reading a research article, students mentioned issues associated with determining the big picture of a study (Table 4: Question 1, 25 percent, Question 2, 5 percent, Question 3, 31 percent). Participants provided suggestions on how to alleviate these issues: 1) professors can help students to determine the big picture of a research article by summarizing the main concepts at the start of discussion (Big picture, sample quote, Table 3), and 2) students can note and highlight summary sentences in articles (quote below). Assisting with understanding the big picture of a study was the top suggestion for increasing student enjoyment of an article (Table 4, Question 4, 36 percent).

**Table 4:** Student perceptions of reading primary literature.

*Comment categories*	*Percent of total comments*
1. How do you feel about reading scientific articles and why do you feel this way? What do you enjoy about reading scientific articles and what do you dislike about reading scientific articles? (44)	
Big picture	25
Scientific Literacy	14
Jargon	11
Lack of knowledge	11
Topic of article matters	11
Time investment	9
Hold students accountable so they read article	7
Lack of confidence	5
Reading more articles is beneficial	5
Dislike certain assessments	2
2. When reading scientific articles if you find yourself confused or lost, why do you feel this way? (19)	
Jargon	42
Cognitive load	16
Wording/sentence structure	16
Lack of confidence	11
Scientific Literacy	11
Big picture	5
3. What’s the greatest difficulty or frustration you experience when reading a scientific article? (16)	
Big picture	31
Time investment	31
Jargon	19
Wording/sentence structure	13
Lack of knowledge	6
4. What can your professor do to facilitate your understanding or enjoyment of a scientific article? (25)	
Big picture	36
Group discussions	16
Hold students accountable so they read article	16
Dislike certain assessments	8
Topic of article matters	8
Jargon	4
Lack of knowledge	4
Reading more articles is beneficial	4
Outside resources to aid understanding	4
5. What do you think is the most beneficial aspect of reading scientific articles? (18)	
Scientific literacy	44
Real life application	28
Reading more articles is beneficial	11
Increases knowledge of topic	11
Big picture	6

The total number of occurrences is shown in parenthesis. The questions that participants were asked are shown in the shaded boxes. Codes are ordered according to prevalence.

*But one thing I do like is most papers I find like*, *like even with long paragraphs with extensive data*, *there’s a summary sentence at the end of [them]*. *Which is*, *I usually highlight those ‘cause it’s like the base of what you need to know and if you need any more information*, *you know it’s above it*, *and you can just read it*.

Interestingly, some students mentioned that their inability to determine how individual findings relate to each other and to the overall big picture of the research article undermined their comprehension:

*Like if I have to read a paper for class*. *I have trouble like*, *what’s really the important thing here*. *Like I get so caught up in the little details*, *than I don’t get like big picture things […] Like what I said it ties back to the like missing big picture things cause I like*, *know all these little details*, *but I don’t know how to connect them*.

Students also reported that issues with jargon, lack of knowledge, and sentence structure impacted their comprehension and satisfaction when reading primary literature. Jargon featured prominently as the top reason students felt they do not understand articles (Table 4, Question 2, 42 percent). It also impacted student enjoyment of reading primary literature (Table 3, Jargon, sample quote). Lack of knowledge also figured among the most prevalent reasons students dislike reading primary literature (Table 4, Question 1, 11 percent). While some participants noted that lack of knowledge of scientific techniques could make reading an article more challenging (see below), others look up things ahead of time to lessen frustration stemming from lack of knowledge (Table 3, Lack of knowledge, sample quote).

*Like by third and fourth year you’ve heard and seen a lot of things*. *You know what*, *you know what*, *if*, *um… electrophoresis*. *Where if you know what that is*, *probably from early on*. *If you have no idea what that is*, *then you have no reference point*, *you have no mental*, *uh visual conception of it is going to be a lot more daunting*.

Some students also stated that the wording and sentence structure of articles could hinder understanding and cause frustration (Table 4, Question 2, 16 percent, and Question 3, 13 percent) because the way they are structured is confusing to them (Table 3, Wording/sentence structure, sample quote). Some students noted that encountering jargon and the lack of knowledge of terms and experimental setup could increase their cognitive load when reading primary literature (Table 3, Cognitive load, sample quote). Lack of confidence, whether about their ability to interpret data (Table 3, Lack of confidence, sample quote) or their overall ability to understand the text (quote below), added to students’ feelings of confusion when reading primary literature (Table 4, Question 2, 11 percent).

*It kinda psyches you out*, *cause you feel like it’s kinda above you*, *when you don’t even get the introduction*, *you know*.

When asked about the elements of primary literature that they dislike or that lead to confusion, students mentioned aspects of scientific literacy (Table 4, Questions 1 and 2; 14 and 11 percent, respectively). Participants mentioned having issues with interpreting graphs (Table 3, Scientific literacy, sample quote) and understanding research design. Some students insightfully identified that one of the most beneficial aspects of reading primary literature is increased scientific literacy (Table 4, Question 5, 44 percent). In the comment below, it is especially interesting to see the student identify the necessity of prior knowledge in relation to the goal of conducting research in the future.

*The*, *the phrasing it of how could I change this if I were to redo the experiment*. *When you get into that point in your life when you’re doing things like this*, *like you have all this background information*. *This is how it’s done in the community*, *this is what I should be shooting for*.

While students acknowledged that the time investment needed to understand a research article could be frustrating (Table 4, Question 3, 31 percent), they reported that being held accountable for reading an article compelled them to try to understand it (Table 4, Question 4, 16 percent). Ways in which instructors held students accountable included discussions with data interpretation (Table 3, Hold students accountable so they read article, sample quote) and using pre-discussion homework (quote below).

*Well [having to answer questions] is kinda like*, *it’s kinda good ‘cause if you read through it the first time you’re just kinda uh*, *getting the idea of it and if it forces you to go back*, *if you really have to understand an article*, *like if it’s really relevant to your topic in class*.

Having group discussions was one of the top approaches students mentioned to help them understand primary literature (Table 3, Question 4, 16 percent). Some participants preferred low-stakes assessments like homework questions to high-stakes assessments like quizzes (Table 4, Question 4, Dislike certain assessments, 8 percent).

While holding students accountable for engaging with the material is important, the level of student interest seems to factor into how much students enjoy reading an article and the amount of effort students dedicate to understanding the material (Table 3, Topic of article matters, sample quote and Table 4, Questions 1 and 4, 11 and 8 percent, respectively). This participant noted that providing students with a choice of articles within a certain topic could make the experience more enjoyable.

*I think maybe if they gave*, *maybe like*, *a couple options [of articles] ‘cause like*, *it like*, *for the bioluminescence*, *we did a bioluminescence one and I really enjoyed it*, *but like*, *if it came down to something like that*, *maybe if they give you a couple of different options in that area*…

Several times during the focus group interviews, students shared that, despite the effort and frustrations associated with the analysis of primary literature, they know that reading scientific articles as part of their coursework is beneficial (Table 4, Reading more articles is beneficial, Questions 1, 4 and 5; 5, 4, and 11 percent, respectively). Several students vocalized the importance of reading primary literature in different courses as a way to become proficient in analyzing and understanding it (Table 3, sample quote). Participants also mentioned that reading primary literature increased their knowledge on a specific topic and helped them to see the real-life applications of concepts they learned in lectures (Table 3, Increases knowledge of topic and Real life application, sample quotes and Table 4, Question 5, 11 and 28 percent, respectively).

### Student motivation for reading primary literature

To determine what motivates participants when reading primary literature, we analyzed our data in light of the achievement goal theory of motivation [37] (Fig 1). This framework includes two goal orientations: 1) mastery, where students work towards achieving competency in a task, and 2) performance, where students are motivated by how they do on a task relative to their peers. Within the performance orientation, students can be motivated by doing better than their classmates (performance approach) or by fear of failure (performance avoidance). We found that most student comments aligned with a mastery goal orientation (Table 5, 64 percent of comments) and one third of comments reflected a performance goal orientation (Table 5, 33 percent). Only one student comment (Table 5, 2 percent) clearly suggested a performance avoidance goal orientation.

**Table 5:** Student focus group comments classified according to the achievement goal theory framework.

Goal Orientation	Total incidences	Percent of total	Sample quotes
Mastery	27	64	Well, what I was going to say was, pretty much what she said for the discussions, like I endorse those greatly because every one of the discussions we’ve had in advanced cell, I’ve come out knowing more, because [of] seeing other people’s perspective.
			Well it’s kinda like, it’s kinda good ‘cause if you read through it the first time you’re just kinda uh, getting the idea of it and if it forces you to go back, if you really have to understand an article, like if it’s really relevant to your topic in class.
Performance (approach)	14	33	I would say you need to be assessed on it, whether it be a quiz or something you turn in, because if not, a lot of people aren’t going to pay attention to it.
			Expecting a quiz or something afterwards really helps me. Like it pressures me to like actually go through the paper, and actually understand it.
Performance (avoidance)	1	2	I have to know this, ‘cause it will reflect poorly forever on my transcript.

Categories are arranged by prevalence.

We also examined student comments in light of the expectancy-value theoretical framework [49] (Fig 1). This framework encompasses students’ expectancy, or expectation of future success in a task, as well as the qualities or values students consider when completing a task. The four task values are attainment, cost, intrinsic, and utility [26, 37, 49, 50]. The balance between these four values helps determine how deeply students connect with a task [49, 50]. We did not find clear indications of student comments related to the expectancy construct of the framework, so we focused our analysis on the value constructs. We found that 37 percent of student comments related to the utility of reading primary literature (Table 6). For example, students in our study found articles to be useful for their future career plans and current research endeavors (Table 6, Utility, sample quotes). The second largest value category was intrinsic, with students in our study more invested in reading articles that appealed to their interests (34 percent, Table 6, sample quotes). While 26 percent of student comments focused on the cost value by indicating that reading complex primary literature is demanding, we were surprised to find that this was not the most prevalent category of comments (Table 6). Finally, only one student made a comment that reflected an attainment value (Table 6, sample quote).

**Table 6:** Student focus group comments classified according to the expectancy-value theory.

Task value	Total incidences	Percent of total	Sample quotes
Utility	13	37	I used to like hate them, or dread them, ‘cause I had such trouble until I started doing actual research, and I could look up papers that I wanted.
			It made me realize that like I love research and I can use my science degree in something that isn’t you know, there are so many pathways that you can take. And yeah, it made, it made me realize I want to do research someday, so I feel like it helps you, you know realize the science behind stuff and what you enjoy out of science.
Intrinsic	12	34	It really is dependent chiefly on am I interested in the specific subject matter there. Cause, cause if it’s something interesting then I can really dive into it. But if I’m being arbitrarily assigned something I don’t care, then it’s going to be very difficult to absorb.
			Well, if I’m not, like, interested in the topic then I usually don’t like it ‘cause, there’s like a bunch of facts and facts like, that […] you don’t really care about or know about it’s just like, what’s the point of this? But when you’re interested in it, it’s nice to know what you’re reading about and I want to know what that means and like, you’re kind of into it and like, figuring it out so it’s kind of fun.
Cost	9	26	I think initially, when I first started reading papers here, I just hated it because it was just, like, really, like super hard words and everything.
			At the end of the day, after you’ve been suffering through physics, organic chemistry, whatever the heck else it is you have to do on this campus and to throw this multiburden paper on top of all that, it’s I’m done, I’m going into communications as a major. So if there was material, and I, I, know it’s part of a whole game, so if there is material that might be a little more demanding, the more explanation, I think, the more preliminary evaluation of it might help in those circumstances. Like, expect to see this, expect to see that. This is a process we’re talking about here…have fun.
Attainment	1	3	Physicians I know are notoriously horrible at understanding primary literature so that’s why I’m really committed to not be a terrible physician and understand drug literature and so I think it’s just good to understand literature even if you’re never going to do research. It just helps you figure out what is and isn’t constituting a good argument in life.

Categories are arranged by prevalence.

## Discussion

The practice of reading and analyzing primary literature is commonplace in college science classrooms. It is popular because it develops important skills in undergraduates, like critical thinking, data interpretation and scientific thinking [4, 5, 7, 8, 51]. However, students may at times struggle with this pedagogical practice. For these reasons, it is important to determine the source of student challenges and what motivates them when reading and analyzing primary literature. In this study, we conducted focus groups of biology undergraduates to elucidate students’ feelings, interests, motivations, and perceived challenges related to reading scientific primary literature.

### Student feelings, interests, and perceived challenges

The results of our study suggest that students encounter issues with jargon, lack of knowledge, and complex writing structure while reading primary literature (Table 4). Some students noted that these issues could increase cognitive load when reading scientific articles (Cognitive load, sample quote, Table 3). This finding aligns with results from think-aloud interviews of students while they read a research article, where we found that they did not manage the cognitive load associated with reading the article as well as faculty [45]. The top student recommendation for professors to help them better understand primary literature was to provide help with determining the big picture of the study (Table 4, Question 4). Several students mentioned that having the instructor provide a summary or evaluation of the overall idea of the article would help them to better comprehend it. This is supported by our recent findings that when reading a research article, faculty summarize portions of the text more often than students and that their summaries more often contain an evaluation or analysis of material [45]. We also found that summarizing was a key way in which faculty managed their cognitive load when reading primary literature [45]. This suggests that the issues with understanding the big picture of a study that students voiced in the focus groups might also be linked to their inability to manage cognitive load. While the rationale, main findings, and implications of a study are summarized in the abstract, this article component often contains highly technical language. In contrast, instructor-generated summaries that connect results to the overall big picture of a study in terms that are easily digestible may aid comprehension by lowering cognitive load and allowing students to build coherence across seemingly disparate study findings.

While students shared that they sometimes lack appropriate scientific literacy skills when reading primary literature (Table 4, Questions 1 and 2), they also emphasized the importance of reading scientific articles in developing these skills (Table 4, Question 5). This is important to note because, as instructors, we may be concerned about implementing a classroom practice that is challenging for students. For this reason, it is reassuring that although students in our study at times found reading primary literature frustrating, they also realized that this practice is beneficial.

### Student responses in light of achievement goal and expectancy-value theories

When evaluating whether to implement a complex task such as reading primary literature into the classroom, it is important to consider student motivation. The goals and values that motivate a student to pursue a classroom activity contribute to important outcomes like persistence and level of engagement [44], reviewed in [37]. For this reason, we analyzed focus group results in light of the achievement goal and expectancy-value theories of motivation (Fig 1) [27, 49]. In our context, student comments predominantly revealed mastery and performance approach goal orientations towards reading primary literature. A mastery goal orientation results in positive academic outcomes, such as engaging more deeply with tasks, preferring challenging work, and feeling more academically successful [36], reviewed in [37]. Moreover, a mastery orientation combined with a performance approach orientation can be as efficacious in learning as a single goal mastery approach [34]. Thus, our findings suggest that our participants’ attitudes and motivations towards reading primary literature align with goals that lead to positive learning outcomes.

The fact that students in our study show mastery and performance approach goal orientations toward reading primary literature suggests that this pedagogical practice fosters positive student behaviors that are conducive to their academic success. While it is possible that the students we sampled happen to have personal goal orientations that elicit positive affect, it is also possible that the framing of the task, in this case, reading primary literature, causes them to shift their orientations. Students who differ in their personal goal orientations can adopt similar situational orientations depending on the goals that are emphasized in the classroom [36], reviewed in [49]. For example, in classroom situations where the instructor emphasizes learning, mastery of skills, and improvement rather than performance and grades, students are more likely to shift to a situational mastery goal orientation [36]. The vast majority of instructors in our department use the analysis of primary literature as a way to develop and strengthen student skills, and we frame reading scientific articles as a process where students can improve skills with practice. It is possible that our department culture of using analysis of literature to gradually develop a set of skills prompts students to adopt the positive goal structures observed in this study. While many instructors in our department use pre-discussion assignments to compel students to read papers thoughtfully [5], we tend not to grade these harshly and instead use them as a way to provide feedback to students. This aligns with findings that undergraduates’ personal goals shift to mastery goals when they perceive an emphasis on enabling their interest in the topic and can turn to performance avoidance when the evaluation associated with a task is perceived as harsh [36]. It is possible that the voluntary nature of our study sample may have led to a population of students who are disproportionately motivated by mastery and performance-approach goals. It would be interesting to explore if students who are interviewed within a required biology course show the same motivations for reading primary literature as those in this study, or if more of those students hold performance-avoidance goals.

Our results also revealed that students consider the different values associated with reading and analyzing primary literature [45] (Fig 1). In our student population, we found that utility and intrinsic values were voiced most often as motivations for reading primary literature. Students noted that reading primary literature is an important practice, but they better engaged with texts of interest to them (Table 6). The cost in time and effort associated with reading primary literature seemed secondary to their utility (Table 6). If students place a high value on a specific task, they are more likely to put more effort into it and engage with more persistence [52–55]. Moreover, high student interest leads to intrinsic motivation, and this in turn is associated with achievement in the sciences [44]. Thus, our findings predict a high level of engagement for the students in our study, as long as the intrinsic value of the article (i.e., their interest in the topic) is present. We did not find any student comments that pertained to the expectancy construct of the expectancy-value theory. Students may not have felt comfortable admitting their challenges in reading primary literature in front of their peers. Alternatively, the focus group questions were not designed with specific theories of motivation in mind, so this could be why data related to student feelings on their expectancy of success is missing from the interviews.

### Implications for practice

Our findings provide insights into the use of reading primary literature in the classroom in like contexts. First, while many instructors are concerned about using this practice because of student resistance, our findings suggest that students find reading primary literature in the classroom useful (Table 6). For this reason, we predict that highlighting the importance of this practice will lead to increased student motivation to engage with research articles. Instructors may want to underscore the utility value of analyzing primary literature in developing skills and deepening the understanding of theory during an initial discussion of this practice. Second, students in our study enjoyed reading primary literature more if they were interested in the topic and if they could choose from a selection of articles. This is supported by findings that classroom practices can be designed to increase situational interest and motivation [44, 56, 57]. In turn, an increase in student situational interest leads to positive academic outcomes like more cognitive engagement [37]. For these reasons, we suggest that instructors provide students with a choice of articles that target the intended learning outcomes and that they let the class choose which article they want to read. Third, students in our study demonstrated the positive goal orientations of mastery and performance approach when reading articles. This could be explained by students who happen to have those goal orientations or by a classroom setting that fosters mastery of skills. While we cannot as instructors control our students’ personal goal orientations, there is evidence that the way we set up our classes can shift students into a mastery goal inclination [37, 56, 57]. Thus, we suggest that instructors frame the analysis of literature as an activity that increases the mastery of students’ skills instead of a task for students to do well in academically. Since a classroom setting that emphasizes mastery goals can lead to increased student enjoyment (reviewed in [37]), it is possible that describing the analysis of literature in this way will also lead to students who are more content and engaged. Our previous findings suggest that giving students reading assignments before discussion of a research article fosters deeper engagement with the text [5]. Students in this study also voiced the importance of being held accountable through assessments and assignments (Table 4). For these reasons, while we believe it is important to frame the discussion of primary literature in a way that emphasizes mastery of skills, it is essential to remember that undergraduates are also performance-motivated, so graded assignments that compel students to engage with the reading are an important component of this pedagogical practice.

In this study, students voiced having issues and needing help with determining the overall idea, or big picture, of an article. On the one hand, providing students with the overall idea of an article prior to discussing it might provide scaffolding that may lower students’ cognitive load. Cognitive load theory predicts that lowering the complexity, or intrinsic load, of an article will allow learners to devote more of their short-term memory to the encoding of new information into existing schemas, thus increasing comprehension [58]. On the other hand, as learners develop more complex schemas, these types of supports may be counterproductive to their learning, a phenomenon termed expertise reversal effect [59, 60]. An alternate approach is to ask students to summarize article sections or the main idea of an article prior to providing an instructor-generated summary. This may lead to productive failure, which sometimes occurs when students attempt a complex task without prior instruction on the topic [61, 62]. While students may not initially succeed at the task, studies show that engaging in productive failure has positive long-term effects on conceptual understanding and transfer [61, 63–65]. In our context, productive failure would involve a generation phase where students produce summaries of a text followed by an instruction phase, where students compare their work to instructor-derived samples to generate more expert-like summaries. It would be interesting to test whether incorporating the practice of productive failure within the analysis of primary literature results in increased comprehension of the text.

### Study limitations and future research

Despite our small sample size (N = 11), insights from qualitative studies such as ours can provide a detailed view into how students approach learning science [66]. Additionally, our results apply more directly to students in our academic context—a 4-year master’s granting institution in the southeastern US. While we believe these initial results contribute important information on the motivations and goal orientations of biology undergraduates towards reading primary literature, expanding our study to include a larger number of students will likely expand the generalizability of our results.

Additionally, we recruited student volunteers through an open call posted on the Biology Department student listserv. It is possible that students who answered the open call did so because they are more interested in reading primary literature. Student responses to a demographic survey (Table 1, S1 File) reveal that study participants had diverse amounts of experience in terms of reading research articles. This suggests that study participants did not self-select in terms of expertise in reading primary literature. However, the possibility still exists that self-selection in terms of interest in reading primary literature might have impacted the perspectives on student motivations obtained in this study. Results from interviews of randomly selected participants will help to further elucidate the different motivations and goal orientations biology undergraduates have towards reading the primary literature. Our findings also leave open the question of whether the students we surveyed happened to have positive personal goal orientations toward reading primary literature or if initial student orientations changed due to the classroom environment. It would be interesting to investigate student goal orientations at the beginning of the term and after students have experienced primary literature analysis to determine if the way we design this learning environment can influence motivation.

In this study, we utilized focus groups to investigate undergraduates’ views on reading primary literature. Focus groups can result in rich insights stemming from the interactions between individuals in the group. The nature of these group interactions can result in information that could be different, but no less valid, than what may be elicited from participants in a one-on-one interview [67, 68]. In fact, the conversations arising during focus groups can draw out aspects of understanding of the topic being discussed that are sometimes not produced during individual interviews [68, 69]. However, there are limitations associated with focus groups that should be pointed out: 1) it might be difficult to obtain insights on highly sensitive subjects, although the “group mind” might sometimes facilitate participation if individuals have similar views on a subject [67, 68], 2) an individual might dominate the conversation, skewing the views obtained from the interview [68, 70], and 3) individuals tend to echo a popular opinion that surfaces during the interview [70]. We do not feel that the benefits and challenges of reading primary literature is a particularly sensitive subject for the students in our study, given that they freely criticized aspects of this pedagogical practice, even in the presence of a professor (MS-T). We addressed the issue of dominant voices by probing less participatory members of the group for their opinions and thoughts. It is possible that the opinions voiced during the focus group exclude less popular views that students did not feel comfortable voicing. Thus, it would be interesting to conduct one-on-one interviews on this topic with a similar population of students to determine their opinions and motivations towards reading primary literature.

## Supporting information

S1 File Participant responses to demographic survey. (XLSX)

S2 File Focus group coding quantification data. (XLSX)

S3 File GPA data for participants and overall biology major population. (XLSX)

S4 File Motivation framework quantification data. (XLSX)
